# Antibiofilm and Antihyphal Activities of Cedar Leaf Essential Oil, Camphor, and Fenchone Derivatives against *Candida albicans*

**DOI:** 10.3389/fmicb.2017.01476

**Published:** 2017-08-03

**Authors:** Ranjith Kumar Manoharan, Jin-Hyung Lee, Jintae Lee

**Affiliations:** School of Chemical Engineering, Yeungnam University Gyeongsan, South Korea

**Keywords:** *C. albicans*, cedar leaf essential oil, biofilm formation, hyphal formation, *C. elegans*

## Abstract

*Candida albicans* can form biofilms composed of yeast, hyphal, and pseudohyphal elements, and *C. albicans* cells in the hyphal stage could be a virulence factor. The present study describes the chemical composition, antibiofilm, and antihyphal activities of cedar leaf essential oil (CLEO), which was found to possess remarkable antibiofilm activity against *C. albicans* but not to affect its planktonic cell growth. Nineteen components were identified in CLEO by gas chromatography/mass spectrometry, and phenolics were the main constituents. Of these, camphor, fenchone, fenchyl alcohol, α-thujone, and borneol significantly reduced *C. albicans* biofilm formation. Notably, treatments with CLEO, camphor, or fenchyl alcohol at 0.01% clearly inhibited hyphal formation, and this inhibition appeared to be largely responsible for their antibiofilm effects. Transcriptomic analyses indicated that camphor and fenchyl alcohol downregulated some hypha-specific and biofilm related genes (*ECE1, ECE2, RBT1*, and *EED1*). Furthermore, camphor and fenchyl alcohol reduced *C. albicans* virulence in a *Caenorhabditis elegans* nematode model. These results demonstrate CLEO, camphor, and fenchyl alcohol might be useful for controlling *C. albicans* infections.

## Introduction

Candidiasis is an opportunistic infection, which affects skin and mucous membranes in a large number of individuals. *C. albicans* is predominantly nosocomial fungal pathogen, and causes complications, such as, mucosal disease and deep-seated mycoses, in immunocompromised individuals (Chandra et al., 2012). The initial stage of infection is adherence, where yeast cells attach to surfaces and form a monolayer, this is followed by morphological transition involving the elongation of hyphae (Silva-Dias et al., 2015; Carradori et al., 2016), which invade tissues and cause infectious diseases (Fox et al., 2015). Biofilms attach to surfaces or interfaces, and are embedded in a matrix of extracellular polymeric substances, and cause various infections (Costerton et al., 1999). Biofilm formation on medical devices and biomaterials, such as catheters, and heart valves, and cause infections, which are often chronic, with high morbidity and mortality rates (Uppuluri et al., 2010; Seddiki et al., 2013). Furthermore, biofilms are inherently difficult to eradicate in cases of implant-associated infections, and thus, new antifungal agents are required to prevent biofilm formation. Matured biofilms form a protein and carbohydrate rich extracellular matrix, which plays a key role in drug resistance (Zarnowski et al., 2014). Similarly, microbial infections associated with biofilms showed increased levels of resistance against most antifungal agents (Pierce et al., 2013). Indeed, *Candida* biofilms are resistant to many antifungal agents (Ramage et al., 2012). Furthermore, biofilms are equally important in the context of multidrug-resistance (Cooper and Shlaes, 2011).

Essential oils have been widely used to developing drugs for the treatment of various diseases, and are easily accessible (da Silva et al., 2016; Myszka et al., 2016; Tutar et al., 2016). Essential oils from wood, bark, leaves, and flowers are used in folk medicine because they represent an inexpensive alternative to modern medicines. In recent times, essential oils have attracted considerable interest due to their antibacterial and antifungal activities presumably related to their complex chemical compositions (Ghanem and Olama, 2014; Shen and Kamdem, 2015). The antimicrobial and antibiofilm activities of several essential oils have been studied against bacteria (Ceylan and Ugur, 2015) and fungi (Abu-Darwish et al., 2016) particularly *Candida* spp. (Peixoto et al., 2017). For instance, essential oils like citronella and cinnamon have been reported to have antibiofilm and anticandida activities (Almeida Lde et al., 2016). Recently, we found cascarilla bark and helichyrsum oil strongly inhibited *Candida* biofilm and hyphal formation (Manoharan et al., 2017). There are no data on antibiofilm activity of cedar leaf essential oil (CLEO) derived from the Cedar of Lebanon (*Cedrus libani*), which is found in Lebanon and south central Turkey. Although, only a small number of studies have examined its antimicrobial activities and chemical composition (Hudson et al., 2011), it has been established that the oil derived from its wood possesses antibacterial (Zrira and Ghanmi, 2016) and antifungal properties (Fahed et al., 2017). Thus, the aim of this study was to determine the chemical composition of CLEO and to investigate its antibiofilm efficiency. In addition, CLEO and its components were evaluated for hyphal growth inhibition using the nematode *Caenorhabditis elegans* as an *in vivo* model. Scanning electron microscopy (SEM) and confocal laser scanning microscopy (CLSM) were used to investigate the effects of CLEO and its components on the morphology, biofilm formation, and hyphal growth of *C. albicans*.

## Materials and methods

### Yeast strain, materials, and growth conditions

The standard *C. albicans* strain DAY185 used in this study was obtained from the Korean Culture Center of Microorganisms (http://www.kccm.or.kr/). Streaking and subculturing of *C. albicans* strain was performed using potato dextrose agar (PDA) or potato dextrose broth (PDB), unless otherwise specified. *C. albicans* strain was preserved at −80°C in 1 ml of PDB supplemented with 30% glycerol stock and, when needed, streaked on PDA plates. Plates were incubated for 48 h at 37°C, and a fresh single colony was then inoculated into 25 ml of PDB and cultured overnight at 37°C. CLEO and cedar wood oil were obtained from Jin Aromatics (Anyang, Gyeonggi Province, Korea) dissolved in ethanol and the final ethanol concentration in the experiments was always kept below 0.1% (vol/vol). All compounds tested (camphor, camphene, camphorquinone, camphorsulfonic acid, fenchone, fenchyl alcohol, α-thujone, borneol, and thymol) were purchased from Sigma-Aldrich (St. Louis, USA) and dissolved at 0.01% (vol/vol) in ethanol. To determine cell growths, turbidities were measured at 620 nm using a spectrophotometer (UV-160, Shimadzu, Japan). Minimum inhibitory concentrations (MICs) were determined using Clinical Laboratory Standards Institute (CLSI) broth dilution method with slight modification (Alastruey-Izquierdo et al., 2015), using 96-well polystyrene plates (SPL Life Sciences, Korea). *C. albicans* cells were inoculated and cultured overnight in PDB medium (dilution 1:100) at varying concentrations (0.1–1%, v/v, or w/v) of tested compounds for 24 h at 37°C. MIC was defined as the lowest concentration that inhibited at least 80% of microbial growth, as assessed by spectrophotometry (620 nm) and confirmed by colony counting. MICs of essential oils and tested compounds are expressed as percentages (v/v or w/v).

### Biofilm assay

Static biofilm formation assays were performed in 96-well polystyrene plates, as previously reported (Lee et al., 2011). Briefly, overnight cultures of *C. albicans* strains were inoculated into PDB (total volume 200 μl) at an initial turbidity of 0.05 at 600 nm and cultured with or without CLEO or its main components at varying concentrations (0.0005 to 0.05%, v/v or w/v) for 24 h without shaking at 37°C. Biofilm formation was quantified after washing three times with H_2_O to remove all non-adherent cells, staining with crystal violet for 20 min, rinsing three times with H_2_O, and extracting the crystal violet with 95% ethanol. Absorbance was measured at 570 nm, and results are presented as the averages of at least six replicates.

### Biofilm metabolic activity—XTT reduction assay

A colorimetric XTT [2,3-bis(2-methoxy-4-nitro-5-sulfophenyl)-2H-tetrazolium-5-carboxanilide sodium salt] reduction assay was performed using established procedures (Ramage et al., 2001; Nett et al., 2011). Briefly, overnight cultures of *C. albicans* strains were inoculated into PDB (total volume 300 μl) at an initial turbidity of 0.1 at 600 nm and cultured with or without camphor or fenchyl alcohol at different concentrations for 24 h without shaking at 37°C. A XTT reduction kit (Sigma-Aldrich, St. Louis, USA) was used to measure the metabolic activity of biofilm cells. XTT and menadione solutions were mixed at 20:1 (v/v) immediately prior to the assay. PBS was then added to XTT-menadione solution (3.76:1 v/v) and 200 μl of this mix was added to each well containing pre-washed biofilms, and incubated in the dark for 3 h at 37°C. The colored supernatant (100 μl) so obtained was transferred to new microtiter plates, and optical densities were measured at 450 nm. Similarly, the metabolic activity of planktonic cells was measured by using culture supernatants.

To determine the planktonic and biofilm cell viability, colony-forming units were examined (Martinez et al., 2010). A 100 μl of planktonic cells were aspirated from culture supernatants and transferred to new microcentrifuge tubes containing 900 μl of PBS. Similarly, biofilms were removed from the bottom of the plates with a sterile tip to dissociate biofilm cells. The samples transferred to tubes containing PBS were mixed gently and serially diluted onto PDA plates, and incubated at 37°C for 24 h, and colony forming units were calculated.

### Component identification by gas chromatograph/mass spectroscopy (GC-MS)

The chemical constituents of CLEO were determined by GC-MS (Jeol JMS 700 mass spectrometer) equipped with an Agilent 6890N GC DB-5 MS fused silica capillary column (30 × 0.25 m i.d., film thickness 0.25 μm). The conditions used have been previously described (Kim et al., 2015). Briefly, electron ionization was performed at 70 eV, helium was used as carrier at 1 ml/min, and temperatures of the GC injector and MS transfer line were 280 and 250°C, respectively. After injections, the GC oven temperature was maintained at 50°C for 2 min, increased to 250°C at 10°C per min, and held at 250°C for 10 min. Diluted samples (1/100, v/v, in methanol) of volume 1.0 ml were injected manually in split-less mode. The relative percentages of selected essential oil components are expressed as percentages. Components were identified using GC retention times on a DB-5 capillary column and by computer matching mass spectra using the Wiley and NIST libraries.

### Confocal laser scanning microscopy of biofilm formation

*C. albicans* cells were cultured in 96-well polystyrene plates (SPL Life Sciences, Korea) without shaking in the absence or presence of CLEO or its components. Planktonic cells were then removed by washing with PBS buffer three times. *C. albicans* cells were stained with carboxyfluorescein diacetate succinimidyl ester (Catalog #: C34554, Invitrogen, Molecular Probes, Inc, Eugene, USA; Weston and Parish, 1990), which is a minimally fluorescent lipophile, which becomes highly fluorescent when its loses its acetyl groups due to the action of esterases in cells. Biofilms were visualized by excitation using an Ar laser at 488 nm (emission wavelength 500–550 nm) under a confocal laser microscope (Nikon eclipse Ti, Tokyo) equipped with a 20 × objective (Kim et al., 2012). Color confocal images were constructed using NIS-Elements C version 3.2 (Nikon eclipse). For each experiment, at least 10 random positions in two independent cultures were examined.

### Observations of hyphae by scanning electron microscopy (SEM)

SEM was used to observe hyphal formation, as previously described (Lee et al., 2014). Briefly, a nylon filter was cut into 0.5 × 0.5 cm pieces and placed in 96-well plates containing 200 μL cells of turbidity 0.05 at 600 nm. Cells were incubated in the presence or absence (untreated control) of CLEO, camphor, or fenchyl alcohol at 37°C for 24 h without shaking. They were then fixed with glutaraldehyde (concentration 2.5%) and formaldehyde (concentration 2%) for 24 h, and post-fixed by treating them with sodium phosphate buffer, osmium, an ethanol series (50, 70, 80, 90, 95, and 100%), and isoamyl acetate. After critical-point drying, cells were examined under a S-4100 scanning electron microscope (Hitachi, Japan) at a voltage of 15 kV and magnifications ranging from x 2,000 to x 10,000.

### RNA isolation and quantitative real-time PCR (qRT-PCR)

For transcriptional analysis, *C. albicans* was inoculated into 25 ml of PDB broth in 250 ml shake flasks at a starting OD_600_ of 0.1, and then cultured at 37°C for 4 h with agitation (250 rpm) in the presence or absence of camphor or fenchyl alcohol (0.01%). RNase inhibitor (RNAlater, Ambion, TX, USA) was added to prevent RNA degradation. Total RNA was isolated using a hot acidic phenol method (Amin-ul Mannan et al., 2009) and further proceed to clean up this RNA with Qiagen RNeasy mini Kit (Valencia, CA, USA).

qRT-PCR was used to determine the transcription levels of hypha- and biofilm-related genes (*HGC1, HYR1, RAS1, SAP1, TEC1, UME6, ECE1, ECE2, RBT1*, and *EED1*) in *C. albicans* treated with or without camphor or fenchyl alcohol. Gene specific primers were used and *ACT1* was used as housekeeping controls (Supplementary Table 1). The qRT-PCR method used has been previously described (Lee et al., 2011). qRT-PCR was performed using a SYBR Green master mix (Applied Biosystems, Foster City, USA) and an ABI StepOne Real-Time PCR System (Applied Biosystems) on two independent cultures.

### Candida infection in the *caenorhabditis elegans* model

To investigate the effects of camphor and fenchyl alcohol on the virulence of *C. albicans*, the nematode *C. elegans* was infected with *C. albicans* as previously described (Manoharan et al., 2017). Briefly, a freshly prepared overnight *C. albicans* culture (100 μl) was spread onto a lawn on PDA plates and incubated for 48 h at 37°C. Synchronized adult *C. elegans* fer-15; fem-1 nematodes were then allowed to feed on the *C. albicans* yeast lawn for 4 h at 25°C. Worms were collected and washed three times with sterile M9 buffer. Approximately 10 worms were then pipetted into single wells of 96-well plates containing PDB medium and treated with the solutions (300 μl) of investigated compounds at final concentrations of 0.01 or 0.001%. For control experiments, M9 buffer was added to medium. For toxicity assays, 10 non-infected worms were pipetted into single wells of 96-well dish containing M9 buffer and solutions of the compounds (300 μl) were added to final concentrations of 0.05, 0.1, or 0.5%. Plates were then incubated at 25°C for 4 days with gentle shaking. Three independent experiments were conducted in triplicate. Results are expressed as percentages of alive or dead worms after 4 days of incubation, and photographs were taken of worms treated at 0.01% using an iRiS™ Digital Cell Imaging System (Logos Bio Systems, Korea).

### Statistical analysis

At least two independent experiments were conducted and results are expressed as means ± standard deviations. The student's *t*-test was used to determine the significances of differences between treated and non-treated samples. Statistical significance was accepted for *p* < 0.05, and significant changes are indicated using asterisks in figures.

## Results

### The effect of CLEO on *C. albicans* biofilm formation

**Figure 1:**
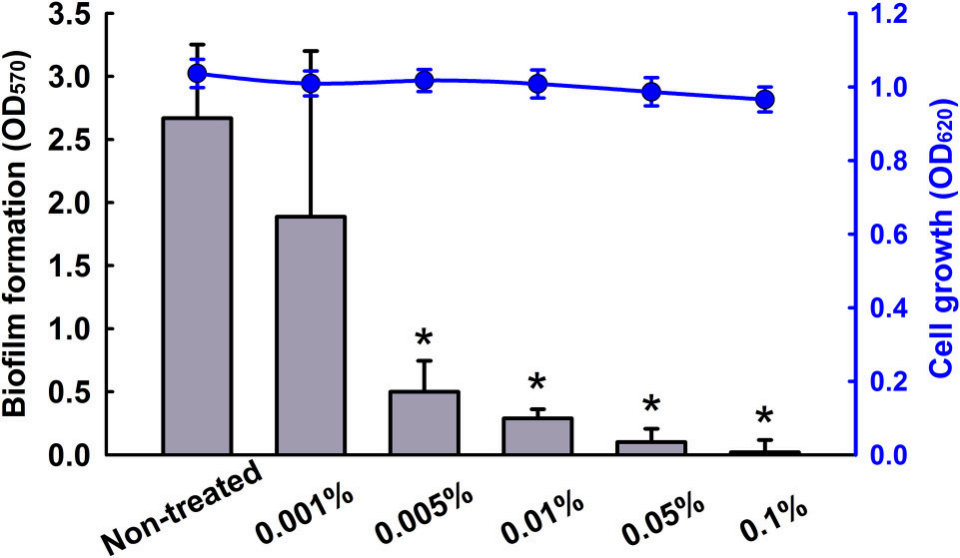
Inhibition of biofilm formation by CLEO. The antibiofilm activity of CLEO against *C. albicans* was determined after culture for 24 h. Two independent experiments were conducted (6 wells per sample); error bars indicate standard deviations. ^*^p < 0.05 vs. non-treated controls.

Previously, we investigated the antibiofilm activities of 83 essential oils against *C. albicans* DAY185 (Manoharan et al., 2017). Of these, CLEO and cedar wood oil at 0.01% inhibited *C. albicans* biofilm formation by more than 85% without affecting planktonic cell growth. Previously, cedar wood oil has been reported to inhibit fungal spores and vegetative cells (Yooussef et al., 2016), and thus, CLEO was examined to determine its antibiofilm activity. CLEO was found to dose-dependently inhibit *C. albicans* biofilm formation (Figure 1), to reduce biofilm formation by 87% at a concentration of 0.01% and completed inhibited biofilm formation at 0.1% (Figure 1). Hence, 0.01% would be considered as reasonable concentration for further study.

### Identification of the main components of CLEO

GC-MS analysis identified 19 compounds in CLEO (Table 1). The major components of CLEO were; fenchone (14.2%), β-thujone (28.0%), α-thujone (20.3%), camphor (8.7%), 1-borneol (4.7%), and endobornyl acetate (4%) (Table 1), which are volatile compounds, mainly monoterpenes. However, a previous study reported different compounds in the ethanol extract of cedar wood and cedar leaf (Loizzo et al., 2008). In particular, himachalene and germacrene were found to be the most characteristics compounds in cedar wood and cedar leaf, respectively. These findings show that the chemical compositions of essential oils vary, and are probably dependent on plant age, soil, and climatic conditions (Polovka and Suhaj, 2010; Kamatou et al., 2012).

**Table 1:** Components of cedar leaf essential oil (*Cedrus libani*) as identified by GC-MS.

**No**.	**SI^a^**	**RT^b^**	**Compounds^c^^,^^e^**	**Composition (%)^d^**
1	840	4.70	1,8-cineole	0.53
2	780	5.56	**Fenchone**	**14.18**
3	866	5.85	**β-Thujone**	**28.01**
4	889	6.01	**α-Thujone**	**20.30**
5	811	6.28	Thujyl alcohol	0.80
6	935	6.43	**Camphor**	**8.66**
7	843	6.66	Isoborneol	0.56
8	888	6.80	**1-Borneol**	**4.71**
9	752	7.13	1- α-Terpineol	2.03
10	570	7.38	Exo-2-Hydroxycineole	0.74
11	603	7.50	D-Fenchyl alcohol	0.77
12	453	7.78	5,6,7,8-Tetrahydro-7-methylidene-5	1.25
13	897	8.46	**Endobornyl acetate**	**4.41**
14	880	8.88	Thymol	3.67
15	488	9.83	Methyl t-5-hydroxy-2-isopropyl-5	1.44
16	646	10.36	Bicyclo	3.12
17	566	10.89	Methyl t-5-hydroxy-2-isopropyl-5	1.44
18	897	15.94	Rimune	1.51
19	815	16.34	Beyerene	1.85

*Components of essential oils present at >4% are indicated by bold font*.

a *SI: Library search purity value*.

b *Retention time (RT)*.

c *Compounds are listed in order of elution from a DB-5 capillary column*.

d *Percentages were calculated by normalizing FID peak areas*.

e *Identification based on computer matching of electron ionization mass spectra using the Wiley and NIST libraries*.

### The antibiofilm effects of CLEO components, camphor, and fenchone derivatives

**Figure 2:**
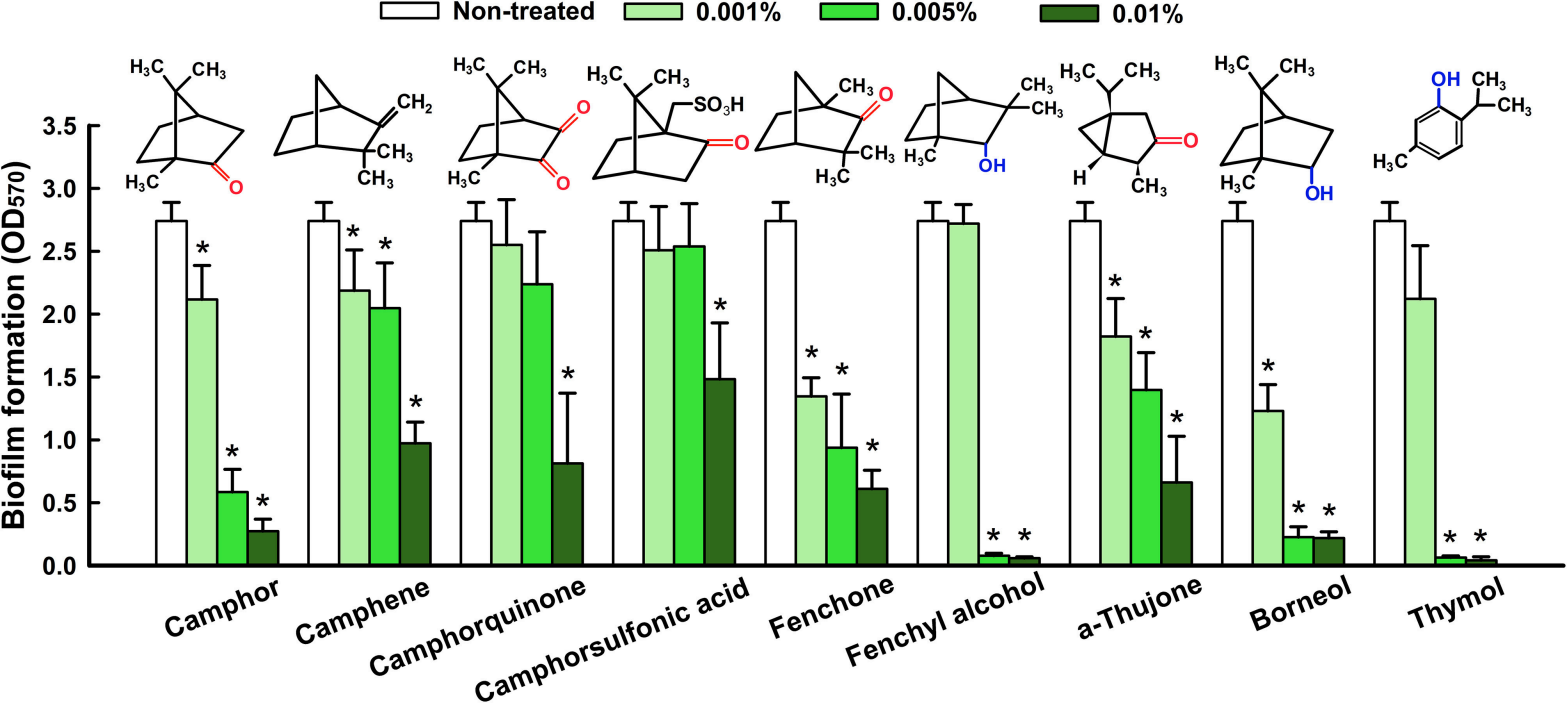
Antibiofilm activity of CLEO components against *C. albicans*. Biofilm formation by *C. albicans* was quantified in the presence of CLEO components after incubation for 24 h. Chemical structures are shown. Hydroxyl groups are shown in blue and oxo- groups in red. At least two independent experiments were conducted (6 wells per sample). Error bars indicate standard deviations. ^*^p < 0.05 vs. non-treated controls.

Antibiofilm efficacies of major components of CLEO and some of their derivatives were examined using *C. albicans*. Nine compounds, namely, camphor, camphene, camphorquinone, camphorsulfonic acid, fenchone, fenchyl alcohol, α-thujone, borneol, and thymol at concentrations of 0.01% significantly inhibited *C. albicans* biofilm formation (Figure 2). In addition, α, β-thujone (mixture of α-thujone and β-thujone) had showed similar activity as α-thujone (data not shown). Consequently, β-thujone could have similar activity against *C. albicans* biofilms. In particular, camphor, fenchone, fenchyl alcohol, borneol, and thymol at 0.005% inhibited biofilm formation by >80% (Figure 2). It appears that combination of camphor and fenchone-related compounds was probably responsible for the observed antibiofilm activity of CLEO. Borneol and thymol have well-known antimicrobial and antibiofilm effects. These compounds have also been found in thymus oils and their activities have been previously evaluated on *Candida* spp. (Khan et al., 2014; Bona et al., 2016). Although, camphor and fenchyl alcohol are components of many essential oils, their antibiofilm activities have not been described, and thus, they were included in the present study.

### Antimicrobial activities of CLEO and its components

**Figure 3:**
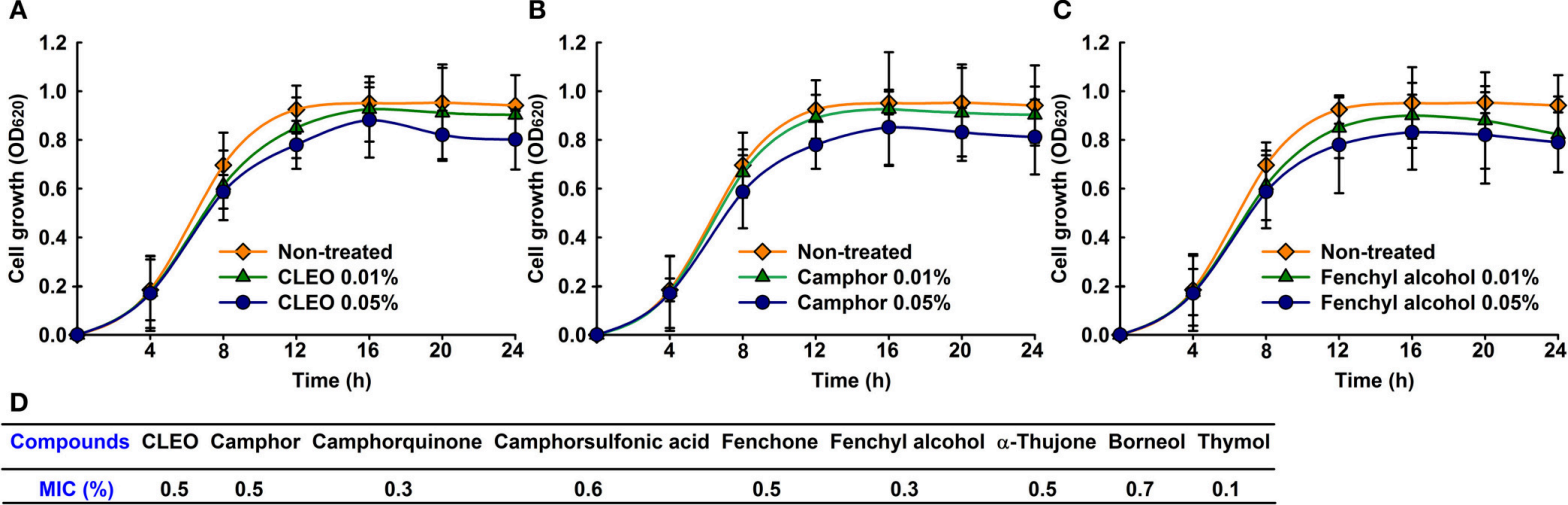
Antimicrobial activities of CLEO, camphor, and fenchyl alcohol. Cell growth of *C. albicans* was measured in the presence of CLEO **(A)**, camphor **(B)**, or fenchyl alcohol **(C)** at different concentrations for 24 h. Minimum inhibitory concentrations of CLEO, camphor, and fenchyl alcohol were determined using a microdilution method **(D)**. At least two independent experiments were conducted.

The cell growth of *C. albicans* was investigated to confirm antibiofilm activities of CLEO (Figure 3A), camphor (Figure 3B) and fenchyl alcohol (Figure 3C). Notably, planktonic cell growth was not affected by CLEO, camphor, or fenchyl alcohol even at concentrations of ≤ 0.05%. The antimicrobial activities of CLEO and its major components were initially evaluated using the microdilution method using *C. albicans* (Figure 3D). The MIC of CLEO was found to be 0.5% (v/v), whereas camphor (w/v) and fenchone (v/v) had MICs of 0.5%. Camphorquinone, fenchyl alcohol, and thymol were more active than CLEO with MICs of 0.1–0.3% (w/v). Interestingly, MIC values were 50-times higher than the concentrations (~0.01%) required for antibiofilm activity. These results confirm that biofilm formation by *C. albicans* was effectively reduced by the antibiofilm activities of these compounds and not by their antimicrobial activities.

### Metabolic activity of camphor and fenchyl alcohol on *C. albicans*

**Figure 4:**
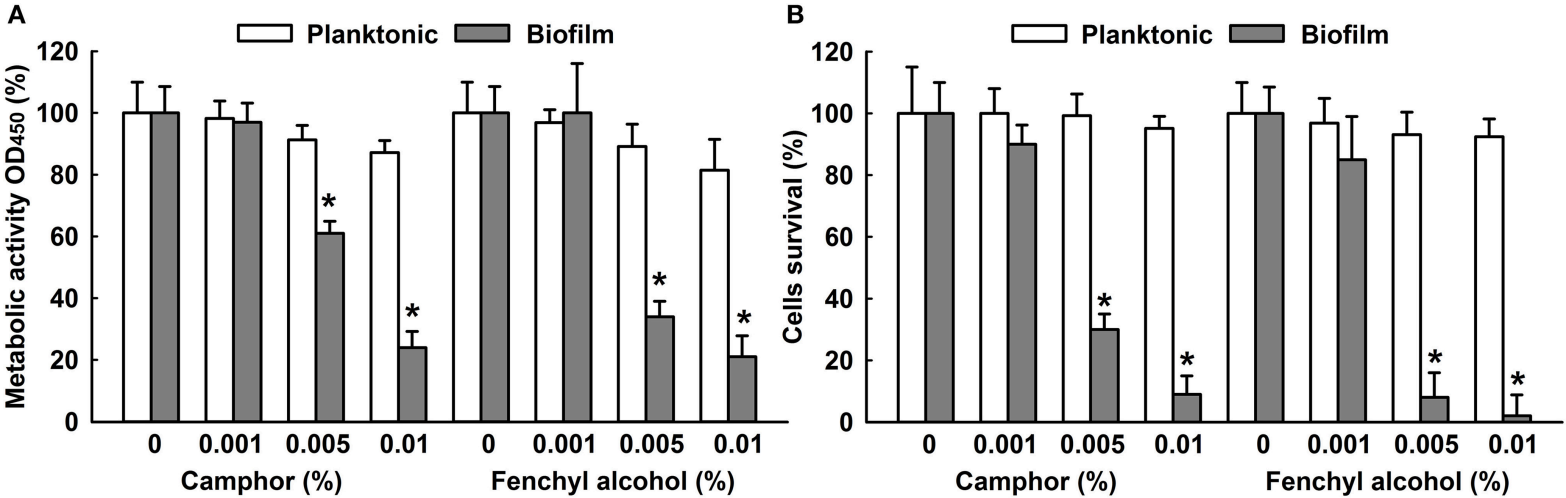
Metabolic activities of camphor and fenchyl alcohol. The effect of camphor and fenchyl alcohol on *C. albicans* biofilms and planktonic cells were quantified using XTT reduction assay and viabilities were calculated by expressing metabolic activities as percentages of non-treated controls **(A)**. The percentage of cell survival of non-treated and treated *C. albicans* cells was measured by determination of the number of colony-forming units **(B)**. Two independent experiments were conducted (6 wells per sample). Error bars indicate standard deviations. ^*^p < 0.05 vs. non-treated controls.

The effects of camphor and fenchyl alcohol on *C. albicans* biofilms and planktonic cells were quantified using XTT reduction assay and cell viabilities. The metabolic activity of *C. albicans* biofilm cells was significantly reduced by 40 and 76% by camphor at 0.005 and 0.01%, respectively. Similarly, fenchyl alcohol reduced the metabolic activity of *C. albicans* biofilms by 66% at the concentration of 0.005% (Figure 4A), and 0.01% reduced biofilms 81%, but no significant effect were seen on planktonic cells. To confirm XTT results, colony forming units were enumerated and expressed as percentage of survival cells (Figure 4B). As expected, biofilm cells were significantly susceptible to 0.005 and 0.01% (>90%) of camphor and fenchyl alcohol (Figure 4B), whereas no significant effect was observed on planktonic cells. These results were consistent with XTT assay and MIC values of camphor and fenchyl alcohol treated cells.

### Hyphae inhibition by CLEO and its components

**Figure 5:**
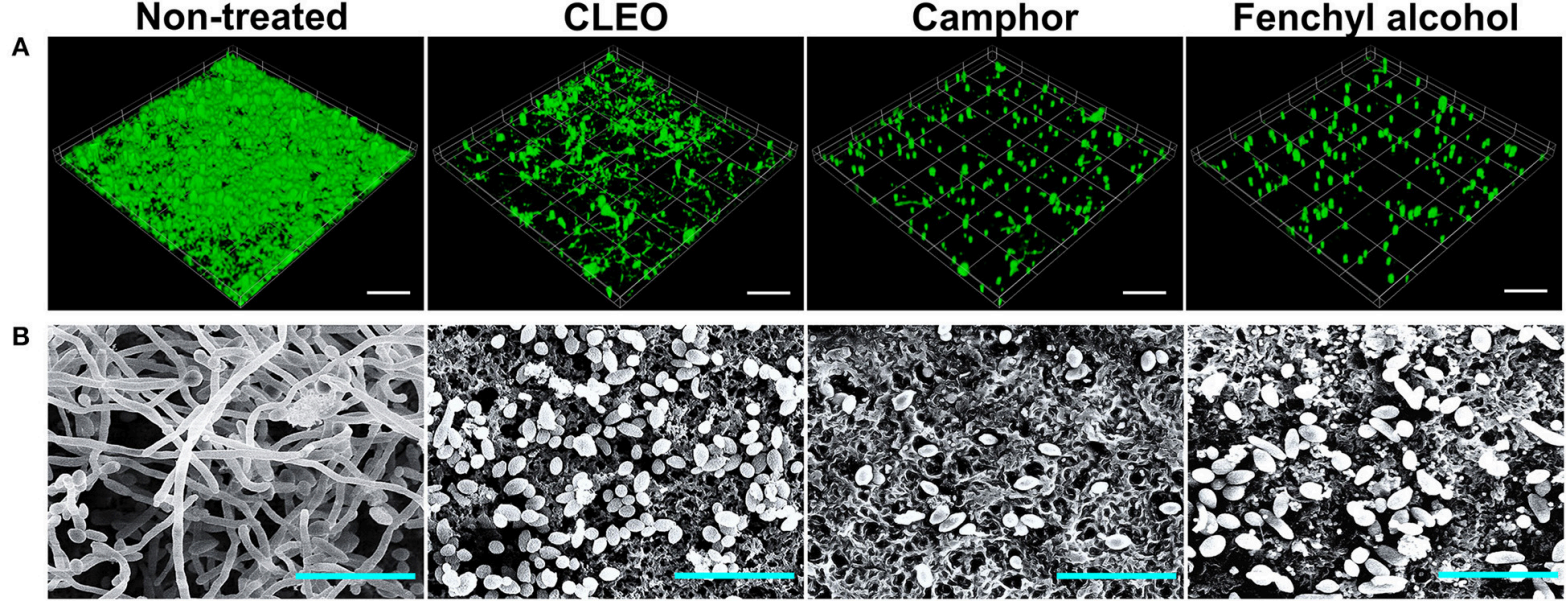
Microscopic observation of the effects of CLEO, camphor, and fenchyl alcohol on biofilms. Biofilm formation by *C. albicans* on polystyrene plates was observed in the presence of CLEO, camphor, or fenchyl alcohol at 0.01% by confocal laser microscopy **(A)**. Scale bars represent 100 μm. Inhibition of hyphal growth was visualized by SEM at a concentration of 0.01% **(B)**. The scale bar represents 20 μm. At least two independent experiments were conducted.

To investigate the effects of CLEO, camphor and fenchyl alcohol on cellular morphology and hyphal growth, we used confocal laser scanning microscopy (CLSM) and scanning electron microscopy (SEM). CLSM images showed that *C. albicans* formed dense biofilms and hyphal cells in non-treated control samples, whereas in the presence of CLEO, camphor, or fenchyl alcohol at 0.01% biofilm adherence and thicknesses were reduced (Figure 5A). SEM revealed that in the non-treated controls, *C. albicans* biofilms were a mixture of yeast cells and hyphae (Figure 5B). However, CLEO, camphor, or fenchyl alcohol at 0.01% inhibited hyphal growth (Figure 5B). Taken together, these results showed CLEO, camphor, and fenchyl alcohol potently inhibited hyphal formation and biofilm formation by *C. albicans*.

### Transcriptional changes in *C. albicans* cells by camphor or fenchyl alcohol

**Figure 6:**
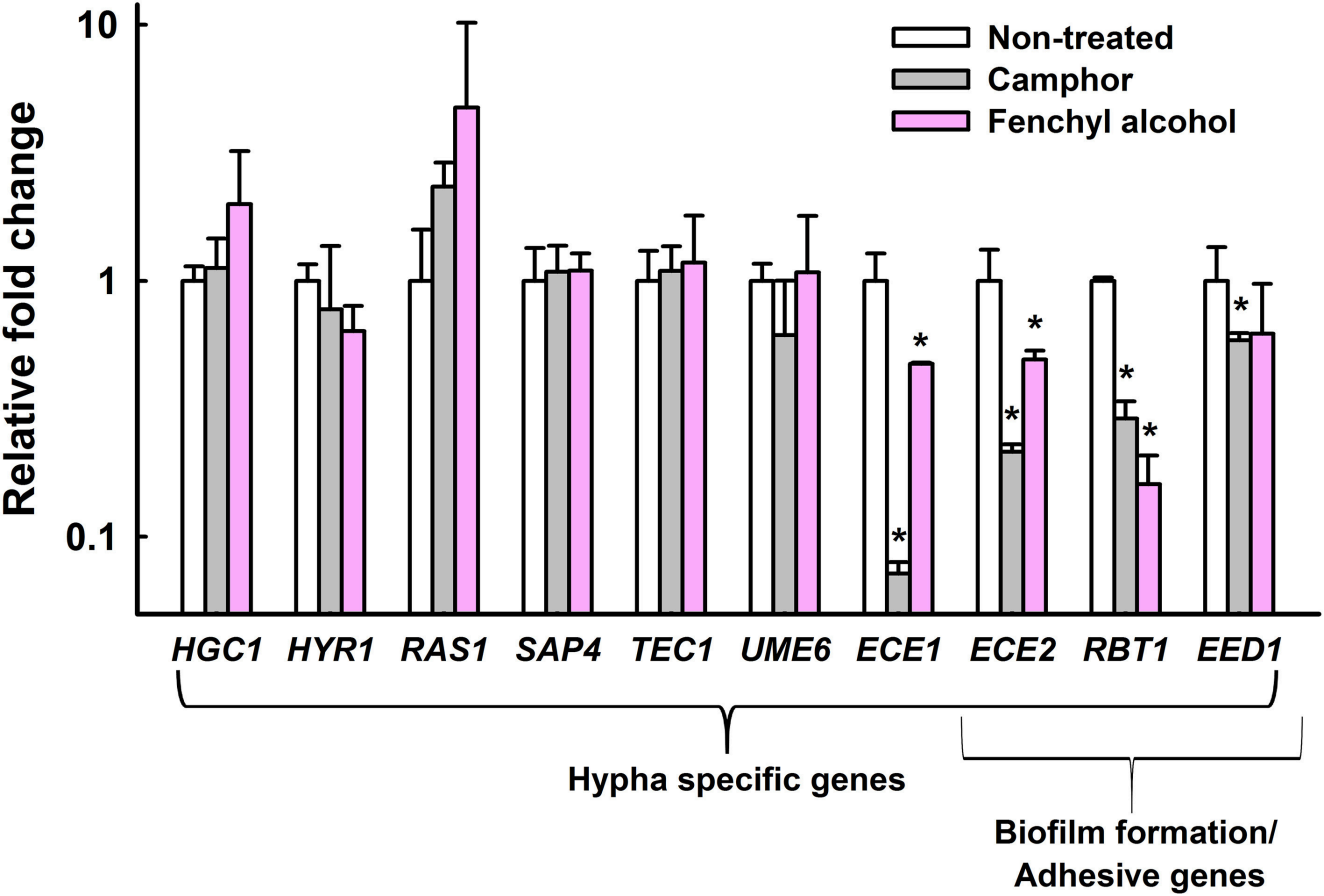
Transcriptional profiles of *C. albicans* cells treated with or without camphor or fenchyl alcohol. *C. albicans* was cultivated with or without camphor (0.01%) or fenchyl alcohol (0.01%) for 4 h. Transcriptional profiles were measured by qRT-PCR. Relative expressions represent transcriptional levels after treatment with camphor or fenchyl alcohol as compared to non-treated controls. Fold changes represents transcription changes in treated *C. albicans* vs. non-treated controls (value of 1.0). The experiment was performed in duplicate. Error bars indicate standard deviations. ^*^p < 0.05 vs. non-treated controls.

To investigate the anti-biofilm mechanism of camphor and fenchyl alcohol, qRT-PCR was performed to examine the expression changes of hypha specific genes and biofilm related genes in treatment and non-treated *C. albicans* cells. Camphor and fenchyl alcohol significantly downregulated the expression of some hypha-specific genes and adhesive related genes. Notably, the expression of hypha-specific gene *ECE1* was downregulated by 14.2-fold after camphor (0.01%) treatment, while adhesive related genes such as *ECE2* (*HWP1*), *RBT1* and *EED1* were also downregulated by 4.7-, 3.5-, 1.7-fold, respectively when compared to their respective controls (Figure 6). Similarly, the expression of *ECE1, ECE2*, and *RBT1* were significantly downregulated by 2.3-, 2-, and 6.2-fold, respectively after fenchyl alcohol (0.01%) treatment. Nevertheless, *HGC1, HYR1, RAS1, SAP1, TEC1*, and *UME6* were not affected after camphor or fenchyl alcohol treatment. Taken together, qRT-PCR results showed that both camphor and fenchyl alcohol affected the expression of some hypha-specific genes and adhesive related genes, supporting the reduction of biofilm formation and hypha production (Figures 2, 5).

### Camphor and fenchyl alcohol inhibited the virulence of *Candida* in *Caenorhabditis elegans*

**Figure 7:**
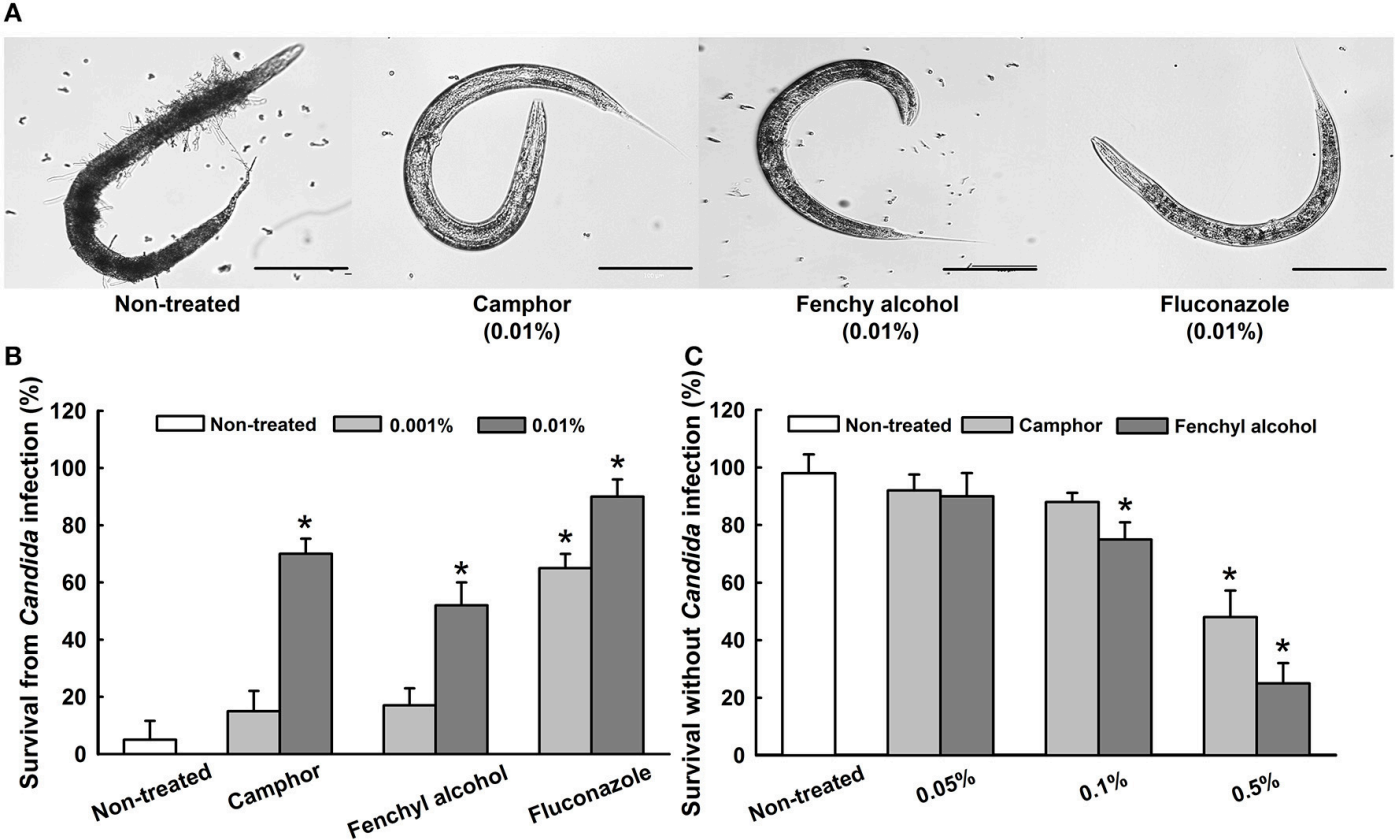
Effects of camphor and fenchyl alcohol on the *C. albicans* infected nematode model of *C. elegans*. Microscopic images of infected nematodes in the presence of camphor or fenchyl alcohol **(A)**. The scale bar represents 100 μm. Bar graph indicates percentage worm survival after exposure for 4 days to essential oil, camphor or fenchyl alcohol **(B)**. The toxicities of camphor and fenchyl alcohol were studied on non-infected nematodes by determining survival rates after 4 days **(C)**. Fluconazole was used as a positive control. Worm survival was determined based on movement. At least two independent experiments were conducted. Error bars indicate standard deviations. ^*^p < 0.05 vs. non-treated controls.

Antihyphal effects of active compounds such as camphor and fenchyl alcohol were examined in *C. elegans* infected with *C. albicans*. Microscopic observations of infected nematodes revealed that *C. albicans* hyphae pierced worms' cuticle and caused death. However, no hyphal formation was observed in worms treated with camphor, fenchyl alcohol, or fluconazole (the positive control) at 0.01% (w/v) (Figure 7A). In *C. elegans, C. albicans* caused a significant decrease in survival (5%) in non-treated controls, whereas >70% of nematodes survived 4 days in the presence of camphor at 0.01%, >50% survived 4 days in the presence of 0.01% fenchyl alcohol (Figure 7B), and >50% survived 4 days in the presence of fluconazole (a commercial antifungal agent).

We also examined the toxicities of CLEO, camphor, and fenchyl alcohol by treating worms with different concentrations for 4 days in the absence of *Candida* (Figure 7C). Camphor and fenchyl alcohol at concentrations of 0.05 and 0.1% did not affect nematode viability or survival (Figure 7C), whereas nematode survival decreased substantially after exposure at >0.5%. These results suggest camphor and fenchyl alcohol at the concentrations administered (< 0.05%) do not have toxic effects on the nematode.

## Discussion

*C. albicans* infections are the most frequently associated with biofilm formation, notably on biomaterials used in clinics (Ramage et al., 2006). The numbers of studies conducted on the properties of essential oils have increased recently due to their potential therapeutic effects against various infections, including candidiasis. In the present study, we focused on the antibiofilm activity of CLEO, which is widely used as a traditional medicine to treat upper respiratory symptoms and wounds, actually, it is also used for embalming. However, many cedar oils are obtained from the family Cupressaceae, particularly from *Thuja occidentalis* and *Thuja plicata*, the oils of which have been reported to have antifungal activities against *C. albicans* and *Aspergillus niger* (Hudson et al., 2011).

The components of essential oils vary between species and between portions of same plants, but they are composed of compounds derived from terpenes and their oxygenated derivatives. The compounds identified in CLEO were mainly monoterpenes and sesquiterpenes with known antimicrobial activities (Ghanem and Olama, 2014; Jasuja et al., 2015). In the present study, CLEO and its components camphor and fenchyl alcohol were found to have anti-candida activity and to exhibit antibiofilm activity (Figure 2). Furthermore, other compounds like borneol and thymol were also found to have significant effects on *Candida* biofilms (Figure 2). It has already been reported that thujone (Dorman and Deans, 2000; Tsiri et al., 2009), fenchone (Gursoy et al., 2009), borneol (Luo et al., 2014), and thymol (Raut and Karuppayil, 2014; Ceylan and Ugur, 2015) which are also components of essential oils have potent antimicrobial activities. Although, camphor and fenchyl alcohol have been previously reported to have antimicrobial activity (Rios et al., 2003; Hua et al., 2014), however this is the first report that the antibiofilm activities (Figure 2) of CLEO, camphor, and fenchyl alcohol involves the inhibition of hyphal growth (Figures 5, 6). In addition, the MICs of thymol found to be higher than those of camphor and fenchyl alcohol, which suggests they are less toxic (Figure 3D).

We also tried to understand chemical structure-activity relationships and to identify the structural motif present in camphor and fenchone derivatives responsible for antibiofilm activity against *C. albicans*. The difference between camphor and borneol and between fenchone and fenchyl alcohol is that camphor and borneol contain hydroxyl (Granger et al., 2005) and oxo-groups, which suggests that hydrogen or carbonyl bonding are responsible for their antibiofilm activities (Figure 2). Although, the chemical structure of camphor is quite different from that of fenchone, particularly with respect to the position of the oxo- group on the aromatic ring, this difference did not appear to influence their antibiofilm activities (Figure 2).

The fact that borneol and fenchyl alcohol contain a hydroxyl group position at similar positions led us to hypothesize that they interact with the same amino acid residues on target cells (Takaishi et al., 2014). Our results suggest that the presence of a hydroxyl group increases the anti-biofilm activities of terpenes, which is supported by the finding that the meta positioned hydroxyl groups of thymol are related to its antimicrobial activity against *Bacillus cereus* (Ultee et al., 2002). Furthermore, it has been reported the antimicrobial activity of terpenes is also dependent on the presence of a hydroxyl group (Koroch et al., 2007). In this previous study, the presence of a hydroxyl group in thymol was found to confer antimicrobial activity against several bacteria.

On the other hand, the presence of two or one oxy- groups with sulfonic acid function in the structure of camphor derivatives decreased the antibiofilm activities of camphorquinone and camphorsulfonic acid (Figure 2). Hydrophobic interactions between compounds and lipid membranes influence membrane integrity and cell permeability (Kim et al., 2007). We hypothesize that hydroxyl groups directly interact with lipids in the extracellular polymeric substances (EPSs) of fungal membranes. It has been previously noted monoterpenes interact easily with the phospholipid membranes of fungi, and thus, damage these membranes and disturb fungal growth (Trombetta et al., 2005; Bakkali et al., 2008; Bendaha et al., 2011), hence it would seem the presence of camphor and fenchyl alcohol in CLEO enable interaction with fungal cell walls, changes cell permeability, and reduces biofilm growth.

Since *C. albicans* is a dimorphic fungus that is capable of switching yeast cells into hyphal cells, which are thought to play an important role in the pathogenesis of fungal infections due to their invasive natures (Sapaar et al., 2014; Sturtevant, 2014). In the present study, CLSM showed that CLEO, camphor and fenchyl alcohol reduced *C. albicans* biofilm formation on surfaces and suppressed virulence factor. Similarly, *Candida* biofilm thicknesses were dramatically decreased by treatments with CLEO, camphor and fenchyl alcohol at 0.01% (Figure 5A). This phenomenon may be caused by the reduction of cell viability, which is positively correlated with XTT reduction and colony forming unit assay results that camphor and fenchyl alcohol at 0.01% reduced biofilm cell metabolism (Figures 4A,B). It was noted that in the present study, extensive hyphae were observed in *C. albicans* biofilms by SEM (Figure 5B), and that all three inhibited hyphal growths. Previous reports have emphasized the importance of hyphal inhibition for reducing the virulence of *C. albicans* (Sudbery, 2011; Nithyanand et al., 2015). Furthermore, we suspect that this inhibition of hyphal growth induced reverse morphogenesis from hyphal cells into the yeast form. Similar results have been reported for sophorolipid and gymeneic acid against *Candida* biofilms (Vediyappan et al., 2013; Haque et al., 2016).

In the transcriptomic study, several hypha-specific genes and adhesion related genes such as *ECE1, ECE2* (*HWP1*), *RBT1*, and *EED1* were downregulated after camphor and fenchyl alcohol treatment (Figure 6). *ECE1* is essential for hyphal development and its expression correlates with cell elongation and biofilm formation (Nobile et al., 2006a). *HWP1* also known as *ECE2*, encodes cell wall mannose proteins that are essential for hyphal development (Nobile et al., 2006b) and intercellular adherence (Orsi et al., 2014). EFG1 is a transcription factor of Ras/cAMP pathway which involves in regulating hypha-specific genes *ECE1* and *ECE2* (Hogan and Sundstrom, 2009). Consequently, these genes were downregulated by camphor and fenchyl alcohol treatment (Figure 6). Another gene, *RBT1* has greater sequence similarity with *HWP1*, required for virulence which rapidly induces filamentous growth as equally as *HWP1* (Braun et al., 2000). Based on these previous reports, the proteins encode by *HWP1* and *RBT1* are almost identical (42% identity in the amino acid sequence) to each other. *EED1* is essential for hyphal formation and its expression involves in the maintenance of hyphal elongation (Zakikhany et al., 2007). Interestingly, these downregulated genes (*ECE1, ECE2, RBT1*, and *EED1*) were regulated in the late phase of Ras/cAMP pathway (Biswas et al., 2007; Tsang et al., 2012; Hsu et al., 2013), hence we hypotheses that camphor and fenchyl alcohol might alter the Ras/cAMP pathway which lead to inhibition of hyphal transition and biofilm formation.

We found that treatment with the CLEO components camphor and fenchyl alcohol at 0.01% significantly increased the survival rate of *C. elegans* in an infection model and prevented the hyphal growth of *C. albicans* (Figure 7). Similar results were observed in earlier studies when nematodes were treated with gymnemic acid (Vediyappan et al., 2013) and linalool or α-longipinene (Manoharan et al., 2017), which effectively prevented the hyphal growth of *C. albicans* within intestines. In addition, the toxicities of camphor and fenchyl alcohol were examined on *C. elegans* and the results obtained revealed that the concentrations required for antibiofilm activity (0.01%) were non-toxic to nematodes. It has been previously reported that natural products, magnolol and honokiol prolonged the survival of nematodes infected by *C. albicans* (Sun et al., 2015). In agreement with this previous finding, our findings indicate that CLEO, camphor, and fenchyl alcohol might be effective for treating candidiasis patients, especially those with implant-associated infections.

We conclude the antibiofilm activity of CLEO is due to the presence of the monoterpenes identified in the present study. To best of our knowledge, this is the first study to provide data on the antibiofilm and antihyphal activities of CLEO, camphor, and fenchyl alcohol against *C. albicans* biofilms. Our results suggest that camphor and fenchyl alcohol have potential use *in vitro* and *in vivo* as antibiofilm agents and demonstrate the ability of CLEO to prevent hyphal formation. In addition, our results show that CLEO, camphor, and fenchyl alcohol are non-toxic to nematodes and suggest they be considered natural alternatives for the treatment of candidiasis patients and medical devices contaminated with biofilms.

## Author contributions

RM and JL performed experiments, analyzed data, and wrote the manuscript. JHL and JL designed the study. All authors have read and approved the final manuscript.

### Conflict of interest statement

The authors declare that the research was conducted in the absence of any commercial or financial relationships that could be construed as a potential conflict of interest.
