# Supplementary material for: Antibiofilm and Antihyphal Activities of Cedar Leaf Essential Oil, Camphor, and Fenchone Derivatives against Candida albicans
Source: Front Microbiol. 2017 Aug 3;8:1476. doi: 10.3389/fmicb.2017.01476 (PMC5541024; doi:10.3389/fmicb.2017.01476)
Supplement: Supplementary file 1 [file Table1.DOCX]

**Supplementary Table 1.** Sequences of the primers used for quantitative RT-PCR

| **Group** | **Gene** | **Function** | | **Primer (5’-3’)** | **References** |
| --- | --- | --- | --- | --- | --- |
| House-keeping | *ACT1* | Actin | | Forward - GCTCCAGAAGCTTTGTTCAGACCAG | This study |
|  |  |  |  | Reverse - GTAATTTCCTTTTGCATACGTTCAG |  |
| Hypha-specific | *HGC1* | Hypha specific G1cyclin related protein | | Forward - GCTTCCTGCACCTCATCAAT | (Hsu et al., 2013) |
|  |  |  |  | Reverse - AGCACGAGAACCAGCGATAC |  |
|  | *HYR1* | Hyphally regulated protein | | Forward - TTGTTTGCGTCATCAAGACTTTG | (Tsang et al., 2012) |
|  |  |  |  | Reverse - GTCTTCATCAGCAGTAACACAACCA |  |
|  | *RAS1* | RAS signal transduction | | Forward - GGCCATGAGAGAACAATATA | (Sun et al., 2015) |
|  |  |  |  | Reverse - GTCTTTCCATTTCTAAATCAC |  |
|  | *SAP4* | Secreted aspartyl proteinase 4 | | Forward - GGTACCGTTGATTTCCAATTC | (Tsang et al., 2012) |
|  |  |  |  | Reverse - ATCTTCACTTTCACGAACACG |  |
|  | *TEC1* | Conserved  filamentation activator | | Forward - AGGTTCCCTGGTTTAAGTG  Reverse - ACTGGTATGTGTGGGTGAT | (Sun et al., 2015) |
|  | *UME6* | Filament-specific transcriptional regulator | | Forward - AGCACCAAATTCGCCTTATG  Reverse - AGGTTGAGCTTGCTGCAGTT | (Hsu et al., 2013) |
| Biofilm/  adhesion | *ECE1* | Extent of cell elongation  protein | Forward - CCAGAAATTGTTGCTCGTGTTGCCA | | This study |
|  |  |  | Reverse - TCCAGGACGCCATCAAAAACGTTAG | |  |
|  | *ECE2 (HWP1)* | Hyphal cell wall protein | Forward - TGGTGCTATTACTATTCCGG | | (Sun et al., 2015) |
|  |  |  | Reverse - CAATAATAGCAGCACCGAAG | |  |
|  | *RBT1* | HWP1 homolog | Forward- CTGCCATTCAACCATCTGCTAACTCCTCATAC | | This study |
|  |  |  | Reverse- GCAGCAAGACCAATAATAGCAGCACCATAAGT | |  |
|  | *EED1* | Epithelial escape and dissemination | Forward - AGCAACGACTTCCAAAAGGA | | (Hsu et al., 2013) |
|  |  |  | Reverse - CGGTTTCTGGTTCGATGATT | |  |
